# Developing and Evaluating a New Preclinical Curriculum with Focus on Prevention in Dentistry

**DOI:** 10.3390/dj13020081

**Published:** 2025-02-14

**Authors:** Ina M. Schüler, Katharina Bach, Pauline Schädlich, Ute Rabe

**Affiliations:** Section of Preventive and Paediatric Dentistry, Department of Orthodontics, Jena University Hospital, 07743 Jena, Germany; katharina.bach@med.uni-jena.de (K.B.); pauline.schaedlich@med.uni-jena.de (P.S.); ute.rabe@med.uni-jena.de (U.R.)

**Keywords:** dental education, teaching methods, dental propaedeutic

## Abstract

**Background/Objectives:** New licensing regulations for dentists in Germany offered the opportunity to modernize the dental curriculum regarding content, methodology and structure. This paper aims to evaluate the newly requested preclinical curriculum “Dental Propaedeutic—Focus on Prevention” by dental students and dental educators and to describe the process of development. **Methods:** The curriculum was developed according to the Kern cycle and the National Competence-Based Learning Objectives Catalog for Dentistry. Lesson planning was based on Bloom’s taxonomy and the taxonomy of significant learning, according to the principle of constructive alignment. A variety of evidence-based teaching methods were implemented. For evaluation, dental students answered a written questionnaire, graded eight topics and added free text. Dental educators were interviewed. **Results:** Thirty out of fifty-four dental students’ enrolled in the third semester participated in the survey, giving the highest grades to the learning atmosphere. All dental educators involved in the delivery of the curriculum participated in the interview. Dental students and educators expressed satisfaction with this comprehensive teaching approach of prevention in an early stage of the dental curriculum. Dental educators reflected on the high workload for development but valued the opportunity to participate in an evidence-based development process and to introduce various modern teaching and examination methods. **Conclusions:** The expenditure of time for the development, performance and examination was high. In order to perform 240 teaching units for eight ECTS credits, 419 h of conceptualization/preparation and 344 h for examination had to be invested. This paper might help to facilitate better understanding of the chances and efforts accompanied by curricular changes.

## 1. Background

In Germany, a new licensing regulation for dentists (https://www.gesetze-im-internet.de/zappro/BJNR093310019.html accessed on 2 December 2024) came into force in October 2020, replacing the previous licensing regulation from 1955.

The new licensing regulations place a strong focus on the prevention of oral diseases, which is reflected, for example, in a required preclinical curriculum with a focus on prevention in dental propaedeutics, which extends over two preclinical semesters. Other significant changes concern the examination system by requesting structured exam questions with predefined expectation horizons in the state dental examinations. Further, the new supervision ratio for students in the clinical courses requires one lecturer to supervise a maximum of three students who treat patients themselves or one lecturer to supervise a maximum of six students during chair-side practical teaching. The requirement for a one-week internship to explore the professional field, a one-month nursing internship and a four-week clinical traineeship was introduced. Finally, several cross-sectional areas and electives were added to the curriculum.

These far-reaching changes offered the opportunity to modernize the dental curriculum, introducing evidence-based teaching and learning methods in a translational approach. As part of preclinical training, the “Dental Propaedeutic—Focus on Prevention” curriculum was designed, implemented and evaluated by the Section of Preventive Dentistry and Pediatric Dentistry at the Dental Clinic of Jena University Hospital.

The paradigm shift to prevention in recent decades impacts dental education [1], defining curriculum frameworks for teaching prevention and population health [2]. Competing with the educational focus on advanced dental technologies and digital dentistry, preventive dentistry and its role in patients’ overall health need to be emphasized [3]. It is argued that preventive dentistry, if placed within the confines of larger courses, might lead to dental students’ under-appreciation of this topic [3]. The weak effect of dental education towards improving dental students’ oral health attitudes was reported in Germany, demanding the implementation of special courses in the dental curriculum [4].

The literature regarding the description of comprehensive preclinical preventive dentistry courses is scarce [3]—yet, several preclinical teaching interventions in the field of preventive dentistry are reported. A French team of dental educators developed a new curriculum on interproximal oral prophylaxis and strongly recommended its implementation [5]. Preclinical training of ergonomic postures is described and recommended [6]. Introducing a teaching module regarding nutrition and food science facilitated dental students’ understanding of the role nutrition plays in oral and general health [7]. Linking preclinical dental education with community-based preventive programs, students are involved in school visits with a near-peer teaching approach [8] or participate in oral health promotion activities in residential facilities for people with intellectual disabilities [9]. Integration of caries prevention in a structured, evidence-based and comprehensive manner into the curriculum was perceived as difficult by faculty due to problems like actual uncoordinated, inconsistent, fragmented teaching of prevention among several departments, lacking support and reinforcement of prevention [2].

To meet the need for comprehensive teaching concepts for prevention in preclinical dental education, this paper pursues two aims: A—to report the development of a preclinical curriculum with a focus on prevention in dentistry and B—to assess the students’ and dental educators’ evaluation and reflection of the new preclinical curriculum. Describing and discussing the development process of the curriculum intends to highlight the chance of implementing various evidence-based teaching and learning methods and the considerable effort involved for the educators and the faculty.

## 2. Methods

### 2.1. Development Process of the Curriculum

The development of the new curriculum followed the steps of the Kern cycle [10], taking into account the principles of constructive alignment [11]. All dental educators involved in the delivery also participated in the development process.

The first step was the problem identification and general needs assessment. Provision of preventive oral care by dentists and empowerment of patients to perform effective oral hygiene regularly in order to keep the oral cavity in good health and function were defined as general needs.

In the second step, the targeted needs assessment, the prevalence of typical oral diseases was analyzed and the extent to which preventive measures are already taught in the dental curriculum was investigated. The prevalence of caries, a preventable disease of the teeth, shows age-dependent values. In Germany, the caries experience grows from 0.5 DMFT (decayed, missed, filled permanent teeth) in 12-year-olds to 11.2 DMFT in adults aged 35–44 years, reaching 17.7 DMFT in younger seniors aged 65–74 years [12]. As many as 10–16% of 3-year-olds and 40–60% of 6- to 7-year-olds are affected by dental caries in the primary dentition [13]. Furthermore, more than half of all adults and almost two thirds of all senior citizens suffer from periodontitis [12]. These rates show that oral preventive measures have to meet a high prevalence of oral disease in all age groups. As caries and periodontal disease have inadequate oral hygiene as a common cause, the aim of the course is to teach risk-oriented oral hygiene strategies adapted to age and needs. Furthermore, the high prevalence of musculoskeletal disorders among dentists [14] testifies to the heavy physical strain of the profession. To counteract this problem, training in ergonomics at the dentist’s chair is needed.

The next step involved defining the learning goals and objectives. The National Competence-Based Learning Objectives Catalog for Dentistry (http://www.nklz.de/kataloge/nklz/lernziel/uebersicht, accessed at 2 December 2024) served as orientation for the content of the curriculum. This describes the knowledge, skills and competencies of dental graduates up to licensure by content and competence level in the sense of a core curriculum. The distribution of content and learning objectives within the different modules of the entire dental curriculum were agreed upon by faculty responsible for the modules.

Educational strategies were developed in step four. The assessment of oral hygiene status and the age- and dentition-specific oral hygiene techniques were to be taught in lectures and in practical sessions through mutual examinations and exercises on models. Ergonomics were planned to be taught theoretically and practically. For oral hygiene training and communication with young children, a kindergarten setting was chosen.

The implementation step required the creation of suitable premises and equipping them with treatment units, instruments and material for the practical teaching elements. Teaching materials, models and equipment were requested from the faculty and ordered. Comprehensive teaching material was developed, procedures for the practical exercises planned and staff trained. The curriculum was planned to take place in the second year of study over the third and fourth semesters.

After the first run of the entire course, an evaluation was carried out. Students’ and educators’ satisfaction was good. As this was the first year of the course, students were specifically asked for feedback on areas for improvement. The information from these evaluations served to optimize individual processes, adapt some content and improve the organization.

The lesson and examination planning was based on Bloom’s taxonomy [15] and the taxonomy of significant learning [16], aiming achieve the highest cognitive level.

Learning objectives, teaching and examinations are aligned according to the principle of constructive alignment [11]. Before each theoretical or practical course, the learning objectives are communicated transparently to the students in order to sharpen the focus on the respective objective during the course and to provide further orientation for self-study. For example, the primary learning objective is assessed in its complexity after several learning units on individualized oral hygiene training as part of the term paper. Here, students develop a prevention plan for a simulated patient, taking into account the dental situation, age, special health issues and restrictions. This prevention plan is accompanied by a videotaped simulated motivational interview. Students received detailed feedback, discussing content and communication aspects.

### 2.2. Evaluation Process of the Curriculum

#### 2.2.1. Assessing Dental Students’ Perspective

All participating dental students (*n* = 54) were invited to evaluate the course anonymously after completion. A simple paper-based questionnaire asked the following questions:

1. How clearly were the learning objectives defined?
2. How suitable were the teaching materials for the topics?
3. How appropriate were the topics?
4. How appropriate was the delivery?
5. How was the time management?
6. How was the gained knowledge?
7. How was the learning atmosphere?
8. What was the overall impression?

The questions were evaluated according to the school grading system familiar to students, with grades ranging from 1—very good to 6—unsatisfactory.

The questionnaire provided space to formulate praise and/or criticism in written free text and dental students were encouraged to communicate their thoughts and ideas on improvement of the course as feedback. The free-text statements were descriptively analyzed by topic.

#### 2.2.2. Assessing Dental Educators’ Perspective

The dental educator responsible for the teaching within the Section of Preventive and Pediatric Dentistry (I.M.S.) interviewed the three dental educators involved in the delivery of the course (K.B., P.S., U.R.).

The years of teaching experience of the dental educators were 19 years (I.M.S.), 14 years (U.R.), 6 years (P.S.) and 4 years (K.B.).

The interviews focused on the dental educators’ reflection regarding the development process, the workload, experiences from the delivery, satisfaction with the performance and observations regarding the students’ behavior and performance. The interviewer took notes during the conversations and described the reflections in the Results section. As co-authors, the interviewed dental educators co-edited the descriptions and assured that their statements and opinions are true and comprehensive.

## 3. Results

The result of the development process was a comprehensive curriculum with defined learning objectives and evidence-based teaching methods.

### 3.1. Learning Objectives

The primary learning objective for the entire curriculum is for students to be able to recommend and justify appropriate age- and needs-related preventive measures. It is not enough for students to be able to recognize and diagnose oral hygiene deficits. Rather, they should be able to select suitable cleaning techniques for the existing oral hygiene deficits and to train the patients, taking into account their general health and motor and cognitive abilities.

Secondary learning objectives include:

The student

- assesses and monitors the oral hygiene situation using plaque indices;
- recommends and justifies appropriate and evidence-based preventive measures for infants and young children, children and adolescents, adults, pregnant women, seniors, patients with malocclusion or orthodontic appliances, patients with fixed and removable dentures or implant-supported dentures, patients with general illnesses (diabetes, cardiovascular diseases, motor and cognitive impairments);
- assesses the patient-related caries risk and derives risk-related preventive measures;
- describes and justifies interactions between oral and general health and their significance in the context of prevention in a simulated patient interview;
- advises on a tooth-healthy diet in a simulated patient consultation;
- provides individualized oral hygiene training with suitable aids (manual and electric toothbrushes, dental floss, interdental brushes, special brushes, fluoride preparations) for the various patient groups;
- assesses the oral hygiene skills of seniors with cognitive and/or motor impairments and derives individualized and practicable prevention strategies;
- performs professional dental cleanings with hand instruments, ultrasound and air flow independently on models;
- implements basic hygiene concepts of dental treatment in a simulated setting;
- operates dental treatment units in a simulated setting;
- implements basic aspects of ergonomics in the dental practice.

### 3.2. Teaching Content and Methods

The teaching consists of lectures and practical courses. One teaching unit takes 45 min.

The lectures cover several subject areas, as presented in Table 1. The topic of oral hygiene was taught in detail with various focal points: biofilm management, de- and remineralization, indices, antibacterial and desensitizing measures. A further topic was on fluorides, their mode of action, dosage forms and toxicology. Using selected studies from current fluoride research, students dealt with basic research methods and critically assessed the study results. Another theme was the role of nutrition for oral health and the interactions between general and oral health. Suitable preventive measures for specific patient groups such as pregnant women, young children, senior citizens and people with special healthcare needs were discussed in several lectures. Lectures also included topics such as halitosis, caries risk assessment, early detection of oral mucosal diseases and malocclusion.

The lecture notes were available to students together with further literature on the Moodle learning platform.

In the lectures, a variety of teaching methods were used. In addition to classic lectures with meaningful case examples from clinical practice, the lecturers used storytelling, plenary discussions, (paired) peer discussions, quizzes and exercises.

In addition to the weekly lectures, students attended practical sessions in small groups, exercising on models and phantom heads as well as on fellow students. Table 2 displays the content and teaching methods of the practical courses. The dates of the practical courses were synchronized by content with the lecture dates.

The students practiced dental plaque assessment with oral hygiene indices and tooth cleaning with rotary instruments, ultrasound and air flow with each other, while the cleaning exercises for the special dentition situations were carried out on models and phantom heads. Oral hygiene training for seniors was practiced with and without the use of geriatric age simulation devices to enable students to experience age-related limitations in motor skills and visual acuity.

A special element of this course was the inter-professional ergonomics training, which was organized together with colleagues from the Occupational Medicine Service and the Institute of Physical and Rehabilitative Medicine at Jena University Hospital. In addition to teaching theoretical content on ergonomics in the dental practice, students were trained in ergonomic positions and movements on dental units under expert guidance.

### 3.3. Structure of the Course

From the students’ perspective, the curriculum comprises 240 teaching units, of which 29 units are lectures without compulsory attendance, 42 units are practical sessions with compulsory attendance and 169 units are self-study. Eight ECTS credits are awarded for the course.

In both semesters, the lectures took place in a lecture hall on a weekly basis without compulsory attendance. The attendance rate of the students was between 60 and 75%.

Each practical course took three teaching units.

The scope of the curriculum from the perspective of the institution was 29 units of lectures and 48 units of practical sessions plus 5 units of group prophylaxis workshops and lectures. The group prophylaxis sessions in the kindergarten were organized and coordinated by the dental educators, but the dental students visited the kindergartens independently in small groups.

The self-study time is calculated in such a way that the students prepare for each lecture for one hour and follow up each lecture for one hour (29 × 2 teaching units = 58 teaching units, corresponding to 43.5 h). For the preparation and follow-up of the practical sessions, two units of preparation and follow-up are planned (7 × 4 = 28 units, corresponding to 21 h). Forty units, equivalent to 30 h, are planned for writing the term paper and forty-three units, equivalent to 32.25 h, for exam preparation.

### 3.4. Control of Learning Success

At the beginning of each practical session, there was a short survey to assess the theoretical preparation with 2–3 questions. The free web-based audience response system Mentimeter (https://www.mentimeter.com/de-DE/features/live-polling last accessed on 2 December 2024) was used online for this purpose. As the results of the knowledge test are immediately visible to all students and the course instructor, any ambiguities or errors were immediately discussed and debated.

A collaborative examination took place at the end of the course. Giving a clinical patient case (paper case), students worked in small groups (2–3 students) to create a risk-oriented comprehensive written prevention plan and filmed themselves in a simulated communication situation with this imaginary patient. The topics of the paper cases are presented in Table 3.

The dental teachers assessed the prevention plans qualitatively and provided elaborate written feedback. In the debriefing sessions, the teachers present the sample solutions drawn up by a panel of experts. After clarifying open questions, the teachers added another element with a potential impact on prevention planning to the respective case description and opened a plenary discussion. Following the principles of the script concordance test [17], the students developed alternative or adapted prevention strategies for the simulated patients, taking into account the added aspects. The teaching expert reinforced the professional exchange of ideas. Adapting the prevention strategy to new conditions aims to activate the students to recall their knowledge and to transfer it critically to the new circumstances. In this way, the development of reflective clinical reasoning and decision making [18] in the students is supported.

The dental instructors assessed the videos of the simulated patient consultations according to content-related and communicative criteria. The students received written feedback on the videos with an assessment of strengths and weaknesses.

As part of the first dental state examination in accordance with the new licensing regulations, students were assessed orally using structured examination forms [19]. The structured examination forms were created by the dental teachers and revised in an expert peer review. They contained a case vignette with a clinical situation and structured questions. Expectation horizons with sample solutions were defined prior to examination. The contents of the sample solutions were divided into three categories in checklist form: answers sufficient for the classification “learning objective achieved” (grade 3), answers that lead to a grade improvement and answers that require a grade deterioration. The clarity of the questions, the informative value of the visual material, the scope of the expectations and the expected answers were discussed by the review board until consensus was achieved. Forty structured examination tasks were created.

### 3.5. Expenditure of Time

The design and creation of the teaching materials took an average of 7 h per lecture and 23 h per practical session and amounted to a total of 387 h (Table 4). The organizational effort for the implementation of the curriculum amounted to approx. 32 h.

The evaluation of the term papers took an average of 90 min per paper, as written formative feedback was given in addition to the summative assessment. Furthermore, on average 30 min per video were needed to review and analyze the interview recordings and provide formative feedback.

It took an average of six hours to create each structured oral examination question. In addition, 20 h were spent revising the examination questions as part of the expert peer feedback, resulting in a total time expenditure of 260 h.

### 3.6. Evaluation of the Curriculum

#### 3.6.1. Students’ Perspective

Of all dental students enrolled in the curriculum (*n* = 54), 55.5% (*n* = 30) took part in the survey. In general, the students were very satisfied, grading the entire course with 1.77. The learning atmosphere was perceived very positively, reaching the highest grades. The lowest grades were given to the issue of time management (Table 5).

The free texts showed that the students (*n* = 12) would have liked more time for the practical exercises, which is also reflected by the low grade for the issue of time management. They criticized the entrance tests without further explanations (*n* = 3). Another point of criticism was the excessive selection of oral hygiene products, which were demonstrated and used in the course (*n* = 2). At the same time, the students were full of praise. They found the supervision and learning atmosphere very pleasant (*n* = 21) and appreciated the opportunity to ask questions and to receive answers patiently (*n* = 16). They described the course as varied (*n* = 15) and informative (*n* = 18). The concurrency between the topics of the lectures and the practical sessions was perceived very positively, as theoretical and practical content were taught in the same period of time (*n* = 16). Students praised the clarity of the instructions (*n* = 11). Particular highlights for the students were the age simulation (*n* = 9), ergonomics (*n* = 20) and professional tooth cleaning with ultrasound and air flow (*n* = 24). The competence and friendliness of the dental educators were also positively emphasized (*n* = 19).

#### 3.6.2. Dental Educators’ Perspective

From the dental educators’ point of view, there was also a high level of satisfaction with the entire course. The collaborative development process was perceived as structured and goal oriented but also time consuming. In particular, the development of the structured exam questions was described as challenging and requiring a high workload. The dental educators were thrilled to select and introduce various teaching methods into the lectures and practical teaching units.

The dental students were perceived as being very interested in the topic of prevention and risk-specific oral hygiene training. The dental educators observed that the aim of the ergonomics training—to raise awareness of the students’ own healthcare—was well reached. The early timing of the entire course in the curriculum is also seen very positively by the teaching staff, so that prevention is taught didactically before invasive treatment.

As expected, there was particular enthusiasm for the first practical dental exercises in the preclinical phase of the course. Due to the heterogeneous prior knowledge of the students—some have completed training as dental assistants—it was sometimes challenging to supervise the small group appropriately, especially with regard to the different pace of work. In general, the educators felt the small group size was beneficial for individual learning support.

Placing the focus on clinical situations, students were repeatedly required to transfer their growing knowledge to specific situations. Here, the lecturers observed a clear increase in the students’ skills.

The educators succeeded in creating a pleasant, learning-promoting atmosphere through a friendly and patient but competent demeanor. The methods used were perceived as suitable and helpful, especially the active learning methods. The educators expressed that the intention is to maintain the variety of methods.

The good results in the students’ final oral examination confirmed that the students have already acquired clinically relevant reasoning and decision-making skills in addition to knowledge and practical skills.

## 4. Discussion

### 4.1. Development of the Curriculum

The process of developing the curriculum “Dental Propaedeutic—Focus on Prevention” was based on evidence following the Kern cycle [10] and respecting the principles of constructive alignment [11].

Mapping the primary and secondary learning objectives of the curriculum also to the Graduating European Dentist (GED) curriculum [20] reveals several conformities. From Domain I [21], learning outcomes regarding professional behavior are covered. Dental students’ competencies to emphasize current concepts of oral health promotion, behavior change and risk assessment, creating personalized methods and approaches required in Domain II [22] are trained within the curriculum. Further conformities exist related to Domain III—patient-centered care [23]—requiring dental students to effectively conduct dietary, behavior and lifestyle analysis, to identify individual risk factors and to develop a comprehensive prevention program for maintaining oral health. This point is the primary learning objective of the course. Dental students’ competencies for maintaining oral health by improving the patients’ oral hygiene regime, providing dietary advice and recommending fluorides stated in the GED curriculum are trained in the course. Some competencies related to Domain IV [24]—dentistry in society—are gained in the course by dental students’ providing oral health promotion in kindergartens, being actively involved in a public health program. First exercises in critical appraisal of published research, defined as the learning objective in Domain V of the GED curriculum [25], are executed by the students on the topic of fluoride toxicity.

A variety of evidence-based teaching and learning methods were implemented in the curriculum. Storytelling, presumably one of the oldest teaching methods that humanity has used [26], appears to have benefits for students’ reflection about their role as dental health professionals [27]. The recently defined term “narrative dentistry” refers to reinforcing patient-centered dentistry through discussing patient narratives [28]. The main goals of storytelling as a teaching method are to facilitate critical reflection among students regarding their professional actions and to balance between objectivity and subjectivity [29]. In the literature, dental students and instructors reported that storytelling was perceived as beneficial for clinical reasoning, engagement, awareness, knowledge acquisition and skill development [30].

The case examples included in the lectures go beyond a simple illustration of the topics taught and tell virtual patient histories. The literature reports that the use of virtual patients has the potential to support the development of clinical reasoning skills [31] and to raise dental students’ comfort in performing operative procedures [32].

The peer and plenary discussions serve to activate the students’ learning process in the setting of a medium-sized classroom [33] and were frequently used in the lectures. The benefit of active or activating learning methods to enhance students’ learning process was emphasized in a recent scoping review [34]. Further, quizzes were used for playful activation [35] and as a simple form of assessment. Short exercises during the lectures also aimed to activate the learning process, for example, by measuring the visual plaque index of the person sitting nearby in the lecture hall. Research suggests that dental students achieve better cognitive performance in preclinical training through the use of audience response systems and that younger students benefited in particular [36]. As it appears that the use of audience response systems only has a minor effect on long-term retention of knowledge [37], they were used primarily as an activating element in the practical courses.

The Peyton method [38,39] was used several times in the practical courses. The effectiveness of this method for procedural skill acquisition and retention was demonstrated, particularly in small group teaching, in a recent systematic review with meta-analysis [40]. The main method for teaching practical skills was based on Halsted’s proven “See One, Do One” [41], even though the Peyton method appears to be superior to the Halsted method [40,42]. During the brushing exercises in the various dentition situations, the students are expected to try out individualized movements for optimized plaque reduction that differ from the classic brushing technique. This individualization as a creative process goes beyond the learning achievable with the Peyton method.

Simulation as an evidence-based, effective teaching method [43] was used in numerous practical courses, for example, the age simulation or the simulation of different treatment postures in ergonomics training. Further, conversational situations with patients of different age groups were simulated with role-playing as a teaching method [44,45].

During and at the end of each practical course, students and instructors were involved in reflection and feedback. Feedback plays a crucial role in education. Elaborated and structured feedback was used in the “Air Flow & Ultrasound” practical course and in the assessment of individual prevention plans [46]. A recent systematic review suggests that feedback contributes to improved psychomotor skills but also points out the limited evidence on how feedback might enhance students’ judgement abilities and performance [47].

Instead of individual written, oral or practical examination at the end of the curriculum, the teacher team decided to implement a written case-based collaborative exam. Research suggests that collaborative testing might enhance the learning process, knowledge retention and teamwork skills [48].

The expenditure of time for the development of the curriculum and the structured examination questions for the final exam is high. To the best of our knowledge, there is no literature reporting that. As the examination questions might be reused in subsequent years with minor adaptation, the high time expenditure for the examinations will be significantly reduced in the future. In addition, the large amount of time spent on conception and preparation will be greatly reduced and limited to updates.

### 4.2. Dental Students’ and Dental Educators Evaluation and Reflection

Both dental students and dental educators report high satisfaction with the curriculum. A comparable teaching approach of preventive dentistry in the preclinical setting reports high knowledge and comfort levels in students who attended a new Introduction to Preventive Dentistry course [3].

The participation rate of 55.5% of dental students in the survey is not very high. This might be caused by the unfavorable time the evaluation was requested, namely after the last teaching unit within the course. Further, the students were encouraged to provide written free-text feedback to express their thoughts on how the course might be improved. This might have contributed to a lower number of students willing to evaluate and to provide more critical comments, pointing helpfully at issues, which might be improved.

Suggestions for improvement from the students’ evaluation are taken into consideration, for example, the stepwise increase of the variety of oral hygiene products in order to avoid the feeling of overwhelm. The amount of time for the practical courses is predefined, so the wish for more time is difficult to fulfill. Regarding the short entrance tests, it was decided to keep them at the beginning of the practical teaching units but to raise the methodological diversity of the tests.

Points for improvement from the dental educators’ reflection include managing the small group composition with respect to prior professional education of the students and to use these resources for peer teaching, to choose multiple and more suitable settings for evaluation and to split the evaluation of the entire course from specific feedback.

## 5. Conclusions

Due to new licensing regulations for dentists in Germany, there was the challenging opportunity to implement a new preclinical course with focus on prevention in the dental curriculum. This paper highlights the chance to introduce a variety of evidence-based methods for curriculum development, teaching and examination in dental education. Despite the high expenditure in time, dental students and dental teachers expressed satisfaction with the concept of teaching prevention comprehensively at an early stage within the dental curriculum. This paper might help faculty and regulatory bodies to understand the chances but also the efforts necessary to implement major curriculum changes.

## Figures and Tables

**Table 1:** Learning Objectives and Teaching methods of the lectures in the Winter Term (WT) and the Summer Term (ST).

Week	Learning Objectives Dental Students…	Teaching Methods
WT 1	… describe the basic scope and aim of age- and needs-related oral healthcare training	Lecture Audience response system Plenary discussion
WT 2	… describe the etiology and epidemiology of diseases affecting the hard dental tissue and the periodontium	Lecture with storytelling Plenary discussion Exercise: “Detecting carious teeth”
WT 3	… explain oral hygiene techniques and devices with focus on biofilm management	Lecture Exercise: “The perfect toothbrush?”
WT 4	… describe and explain concepts of ergonomic workplace design and behavior	Lecture with case examples Plenary discussion Quiz
WT 5	… explain the pharmacology of fluoride and describe products by content and concentration	Lecture Peer discussion in pairs
WT 6	… explain evidence-based fluoridation measures, toxic effects and actions required by overdose	Case-based lecture Storytelling Plenary discussion
WT 7	… explain effects of oral hygiene measures and products with focus on de- and remineralization	Lecture Plenary discussion One-minute paper
WT 8	… explain the assessment of oral health status with different indices and describe monitoring	Lecture Plenary discussion Exercise: “Visible plaque index”
WT 9	… discuss the interactions between oral health and diet	Lecture Plenary discussion Peer discussion in small groups
WT 10	… explain and apply caries risk assessment and derive consequences	Case-based lecture Exercise: Caries risk assessment in three cases Plenary presentation and discussion
WT 11	… describe and explain evidence-based recommendation of preventive measures for infants (aged 0–3 years)	Case-based lecture Peer discussions in pairs One-minute paper
WT 12	… recommend and justify evidence-based preventive measures for children and adolescents (aged 4–20 years)	Case-based lecture Storytelling Reflection on short videos
WT 13	… explain prevention and management of halitosis	Lecture Plenary discussion
WT 14	… describe and explain home-based oral healthcare measures to patients	Lecture Quiz
WT 15	… explain prevention and early detection of malocclusion	Lecture with case examples
ST 1	… explain the role of general health for oral health	Lecture with case examples
ST 2	… discuss the role of oral health for general health and quality of life	Lecture with case examples
ST 3	… recommend and justify preventive measures for seniors with and without special healthcare needs	Case-based lecture
ST 4	… describe the content of structured and comprehensive planning of prevention	Lecture Moodle instructions with examples
ST 5	… recommend and justify evidence-based preventive measures for patients with general diseases	Case-based lecture
ST 6	… explain prevention and early detection of oral mucosal diseases	Case-based lecture
ST 7	… recommend and justify evidence-based preventive measures for pregnant women	Zoom lecture with case examples
ST 8	… recommend and justify evidence-based preventive measures for patients with special healthcare needs	Case-based lecture Plenary discussion
ST 9	… describe and explain the use and effects of antibacterial and desensitizing products	Lecture Plenary discussion
ST 10	… appraise critically examples of scientific literature and evaluate scientific evidence on fluorides	Journal club Students’ presentations Plenary discussion
ST 11	… describe possible examples of sustainability in dental practice	Lecture Quiz Videos
ST 12, 13	… reflect on and discuss their ability to develop an “individualized prevention plan” (Feedback to students’ term papers)	Plenary discussion Script concordance test

**Table 2:** Learning Objectives and Teaching Methods of the practical courses in the Winter Term (WT) and the Summer Term (ST).

Week	Learning Objectives Dental Students…	Teaching Methods
WT 1–3	… utilize the dental units and apply clinical hygiene requirements	Audience response system Peyton Reflection and feedback
WT 4–6	… exercise the ergonomic design of the working place; execute ergonomic postures and show awareness of own musculoskeletal healthcare	Audience response system Demonstration and supervised exercise of postures Training exercised for long-term musculoskeletal health Reflection and feedback
WT 7–9	… demonstrate oral health assessment with various indices, interpret the values and derive oral healthcare recommendations	Audience response system Demonstration and supervised mutual exercises Communication training, role-play Reflection and feedback
WT 10–12	… demonstrate oral health assessment in different dentitions, interpret the values and derive oral healthcare recommendations	Audience response system Demonstration and supervised exercises on models and phantom heads Communication training, role-play Reflection and feedback
ST 1–3	… execute oral health assessment in dentitions with orthodontic devices or malocclusion, interpret the values and derive oral healthcare recommendations	Audience response system Demonstration and supervised exercises on models with malocclusion and orthodontic devices Communication training, role-play Reflection and feedback
ST 4–6	… demonstrate oral health assessment in incomplete dentitions and dentures, interpret the values and derive oral healthcare recommendations	Audience response system Demonstration and supervised exercises on models with incomplete dentitions and different types of dentures Communication training, role-play Reflection and feedback
ST 7–9	…execute oral health assessment in incomplete dentitions and dentures, interpret the values and derive oral healthcare recommendations simulating advanced age	Audience response system Demonstration and supervised exercises on models with incomplete dentitions and different types of dentures with age simulation elements (motor and visual impairment) Communication training, role-play Reflection and feedback
ST 10–12	… demonstrate professional tooth cleaning with air flow and ultrasound	Peyton Demonstration and supervised exercises on models and on fellow students Reflection and feedback

**Table 3:** Topics of the prevention plans and supplemented elements for script concordance testing.

Topic of the Prevention Plan	Supplemented Elements for Script Concordance Testing
Infant with early childhood caries Knowledge deficit of the mother	Blindness of the infant
Teenager with developmental defects of enamel Pain	Asthma of the child
Teenager with fixed orthodontic device Motivational deficit	Epilepsy of the child
Female patient Dental phobia	Pregnancy, eating disorder
Patient with implant denture Motivational and procedural deficit	Endocarditis risk
Senior patient with removable dentures Complex deficits	Dementia

**Table 4:** Expenditure of time needed for conceptualization, preparation, performance and examination expressed in hours (h) and teaching units (tu); 1 tu = 45 min.

Conceptualization and Preparation		Performance/Delivery		Examination	
Creating lectures	203 h	Lectures	29 tu	Evaluation of term paper	27 h
Creating practical sessions	184 h	Practical sessions	144 tu	Evaluation of videos	9 h
Organizational load	32 h	Group prophylaxis	5 tu	Creating structured exam questions	260 h
		Organizational load	20 tu	Organization of the exams	16 h
				Conducting oral examinations	32 h
**Total:**	**419 h**	**Total:**	**198 tu**	**Total:**	**344 h**

**Table 5:** Dental students’ evaluation of the course using the school grading system (1 = very good to 6 = unsatisfactory).

Question	Number of Responses	Median	Mean	Standard Deviation
1—Learning objectives	30	2	1.84	0.69
2—Teaching material	30	2	1.90	0.70
3—Topics	30	2	2.13	0.96
4—Course delivery	30	2	1.74	0.63
5—Time management	30	3	2.48	1.00
6—Knowledge gain	30	2	1.94	0.85
7—Learning atmosphere	30	1	1.16	0.45
8—Overall impression	30	2	1.77	0.56

## Data Availability

Data are contained within the article.
